# 

**Published:** 1923-04

**Authors:** 


### Contents

Event and Comment....................145
Quartz Light (Ultra Violet Radiation)....................147
The Clinical, Pathological and Radiological Aspects of Infection of the Teeth and Gums....................158
United States Civil Service Examination....................175
Indents....................179
Announcements....................184

